# The *Gastrodia menghaiensis* (Orchidaceae) genome provides new insights of orchid mycorrhizal interactions

**DOI:** 10.1186/s12870-022-03573-1

**Published:** 2022-04-07

**Authors:** Yan Jiang, Xiaodi Hu, Yuan Yuan, Xuelian Guo, Mark W. Chase, Song Ge, Jianwu Li, Jinlong Fu, Kui Li, Meng Hao, Yiming Wang, Yuannian Jiao, Wenkai Jiang, Xiaohua Jin

**Affiliations:** 1 Institute of Botany, Chinese Academy of Sciences, Xiangshan, Haidian, Beijing, 100093 China; 2 Novogene Bioinformatics Institute, Beijing, 100083 China; 3 National Resource Center for Chinese Meteria Medica, Chinese Academy of Chinese Medical Sciences, Chaoyang, Beijing, 100700 China; 4 Jodrell Laboratory, Royal Botanic Gardens, Kew, Richmond, TW9 3DS Surrey UK; 5 Department of Environment and Agriculture, Curtin University, Perth, WA Australia; 6 Xishuanbanan Tropical Botanical Gardens, Chinese Academy of Sciences, Menglun, Mengla, Yunnan China

**Keywords:** *Gastrodia*, Genome evolution, Mycoheterotrophy, Mycorrhizal roots

## Abstract

**Background:**

To illustrate the molecular mechanism of mycoheterotrophic interactions between orchids and fungi, we assembled chromosome-level reference genome of *Gastrodia menghaiensis* (Orchidaceae) and analyzed the genomes of two species of *Gastrodia*.

**Results:**

Our analyses indicated that the genomes of *Gastrodia* are globally diminished in comparison to autotrophic orchids, even compared to *Cuscuta* (a plant parasite). Genes involved in arbuscular mycorrhizae colonization were found in genomes of *Gastrodia*, and many of the genes involved biological interaction between *Gatrodia* and symbiotic microbionts are more numerous than in photosynthetic orchids. The highly expressed genes for fatty acid and ammonium root transporters suggest that fungi receive material from orchids, although most raw materials flow from the fungi. Many nuclear genes (e.g. biosynthesis of aromatic amino acid L-tryptophan) supporting plastid functions are expanded compared to photosynthetic orchids, an indication of the importance of plastids even in totally mycoheterotrophic species.

**Conclusion:**

*Gastrodia menghaiensis* has the smallest proteome thus far among angiosperms. Many of the genes involved biological interaction between *Gatrodia* and symbiotic microbionts are more numerous than in photosynthetic orchids.

**Supplementary Information:**

The online version contains supplementary material available at 10.1186/s12870-022-03573-1.

## Background

Orchid family is among the largest plant families with approximately 27 000 species in 750 genera [1]. The germination of dust-like seeds depends on mycorrhizal fungi for nutrients, including organic carbon (C), phosphorus (P) and nitrogen (N) [2, 3]. With plants becoming autotrophy by photosynthesis, heterotrophic orchid seedlings switch to autotrophic adults. Many plants have maintained the ability to live on fungal carbon and gradually lost the capacity to photosynthesize, and these groups range from partially photosynthetic green species to obligate mycoheterotrophs that completely lack chlorophyll and are fully dependent on their fungal associates [4–9].

It is estimated that there are approximately 47 independent origins of full mycoheterotrophy in land plants [10]. Three major fungal lineages, i.e., Ascomycota, Basidiomycota and Glomeromycota, have been involved in the mycoheterotrophic interactions, out of which the Glomeromycota supports the greatest number of fully mycoheterotrophic species [11–13]. The evolutionary dynamics and genetic composition of plant–fungus interactions are largely unknown [14–18].

*Gastrodia* (Orchidaceae; Epidedroideae) comprises ~ 100 species distributed in the Old World subtropical and tropics [10, 19–22] and is the largest genus of orchid obligate mycoheterotrophs. Like most orchids, *Gastrodia* species depend on fungi for seed germination and initially their source of organic carbon, but in *Gastrodia* and relatives (tribe Gastrodieae) this dependence continues throughout their life cycle [4, 23–25]. Compared with photosynthetic orchids, the species of *Gastrodia* exhibit massive changes in their body plans and consist of solely leafless swollen stems (tubers) [9, 14, 24]. Most species of *Gastrodia*, such as *G. menghaiensis* (Supplementary Figure S1a-b), form well-developed mycorrhizal roots, whereas other species, such as *G. elata* (Supplementary Figure S1c), are rootless with their fungal associate directly connected to their tubers [14, 26, 27]. To date, *G. elata* has the smallest known angiosperm genome, containing approximately 18,969 protein-coding genes [9, 28] (but see [29]) with some genes families associated with its mycoheterotrophic lifestyle, such strigolactone signaling and digestion of hyphae, expanded.

These features make *Gastrodia* an important model to study plant–fungus interactions and obligate mycoheterotrophy. Here, we present a high-quality chromosome-level assembly of the *G. menghaiensis* genome and demonstrate that the *G. menghaiensis* genome has experienced massive alterations of the number and kinds of genes. We have found that many of the genes involved biological interaction between *Gatrodia* and symbiotic microbionts are more numerous than in photosynthetic orchids.

## Results and discussions

### Assembly, annotation of genome of *Gastrodia menghaiensis*

**Fig. 1:**
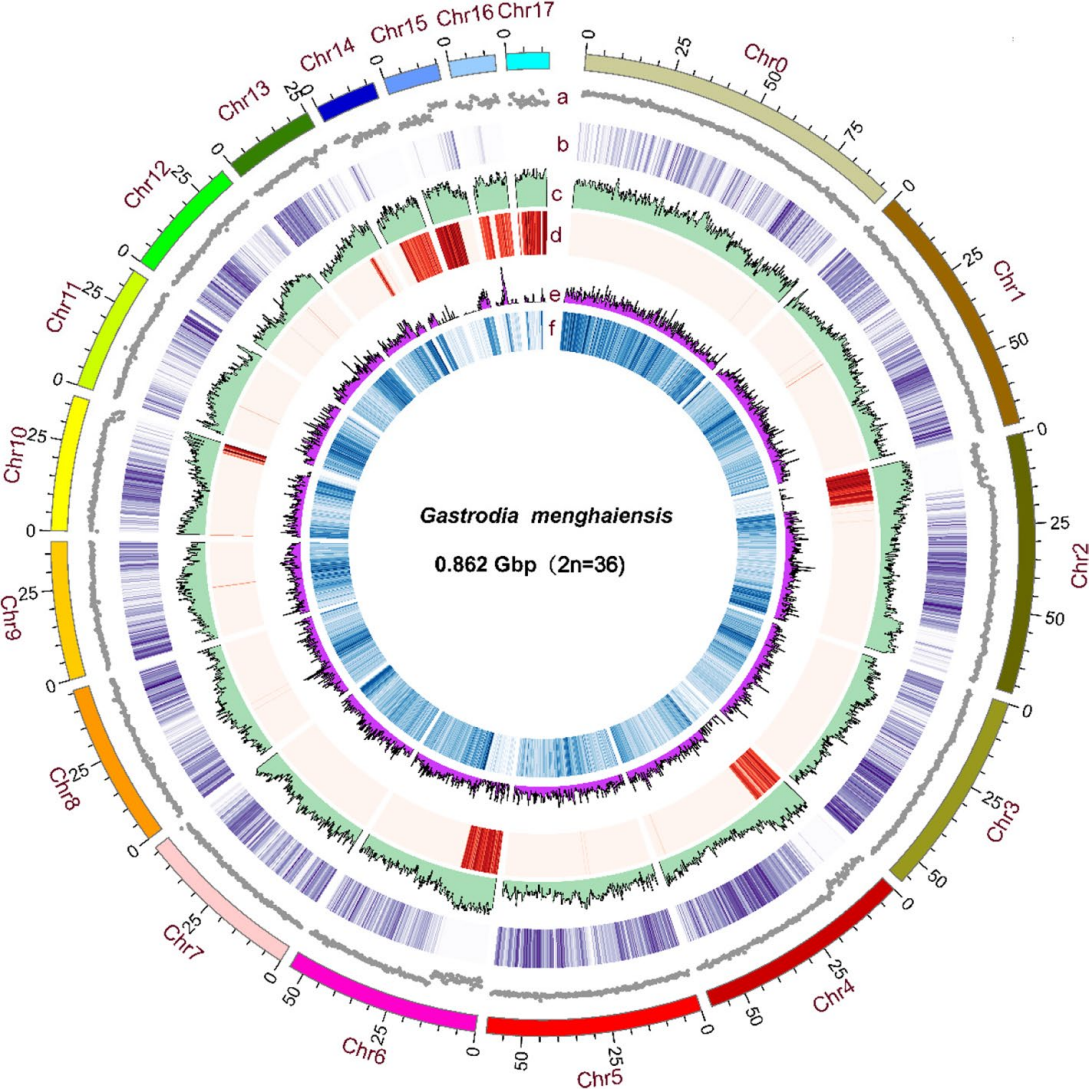
Genome characteristics of *G. menghaiensis*. Track a-f: **a** chromosome, **b** GC density (gray), **c** gene density (purple), **d** transposon element density (green), **e** transposon element density (red), **f** LTR-Copia density (pink), **g** LTR-Gypsy density (blue). All were drawn in a window size of 300 kb, chromosomes units = 1,000,000 bp

The k-mer-based genome size estimate of *G. menghaiensis* is 0.987 Gb with a low level of heterozygosity (0.1%) and high repeats (65.08%) (Supplementary Table S1, Supplementary Figure S2). Whole-genome shotgun sequencing was performed with the PacBio Sequel platform (~ 102.70 × coverage), Illumina Hiseq X-ten (read length of 150 bp, ~ 122.50 × coverage) and 10X Genomics (~ 131.90 × coverage) (Supplementary Table S2). Finally, the assembly consisted of 1,595 scaffolds, with a scaffold N50 of 6.82 Mb (total length = 862.84 Mb) and contig N50 of 2.37 Mb (total length = 859.12 Mb) (Supplementary Table S3). Overall, our results showed that 97.66% of the raw sequence reads could be mapped to the assembly, suggesting that our assembly was nearly complete (Supplementary Table S4). This was further assessed using EST (Expressed Sequence Tag), CEGMA (conserved core eukaryotic gene mapping approach), BUSCO (benchmarking universal single-copy orthologs analysis) [30] and transcriptome data. Approximately 99.8% ETS sequences are covered by our assembly; 232 of 248 (93.55%) conserved core eukaryotic genes from CEGMA were captured in our assembly, and 212 (85.48%) of these were complete (Supplementary Table S5 and S6). BUSCO revealed that 1046 of 1440 (72.7%) highly conserved genes were captured in our assembly (Supplementary Tables S7 and S8). We further revised the *G. menghaiensis* genome assembly using high-throughput chromosome conformation capture (Hi-C) data. The full genome comprises 1506 scaffolds with a scaffold N50 of 54.12 Mb, and 785.36 Mb of the assembly were distributed across 18 chromosome-level pseudomolecules (Fig. 1, Supplementary Tables S3, S9 and S10).

We annotated 539.84 Mb of repetitive elements occupying 62.57% of the *G. menghaiensis* genome (Supplementary Table S3). The majority of the repeats are long terminal repeats (LTRs), about 49.49% of the genome (supplementary tables S11 and S12). Based on a combination of homology search, de novo prediction, and RNA sequence-aided prediction, 17,948 protein-coding genes (PCGs) were annotated with an average length of 13,657 bp (Supplementary Figure S3, Supplementary Table S13). Additionally, 16,402 (91.39%) PCGs were supported by at least one of the transcriptome datasets from tubers, flowers, flower buds and fruits, indicating a high level of annotation accuracy (Supplementary Table S14). The statistics results show that each gene contains 5.15 exons with an average length of 221.78 bp (Supplementary Table S14). Approximately 84.4% of PCGs were functionally annotated by similarity searches against homologs sequences and protein domains (Supplementary Table S15). In addition, we identified noncoding RNA (ncRNA) genes in *G. menghaiensis*, including 157 rRNA, 292 tRNA, 191 miRNA, and 2725 snRNA genes (Supplementary Table S16).

### Extensive loss of genes and gene families in *Gastrodia menghaiensis* genome

**Fig. 2:**
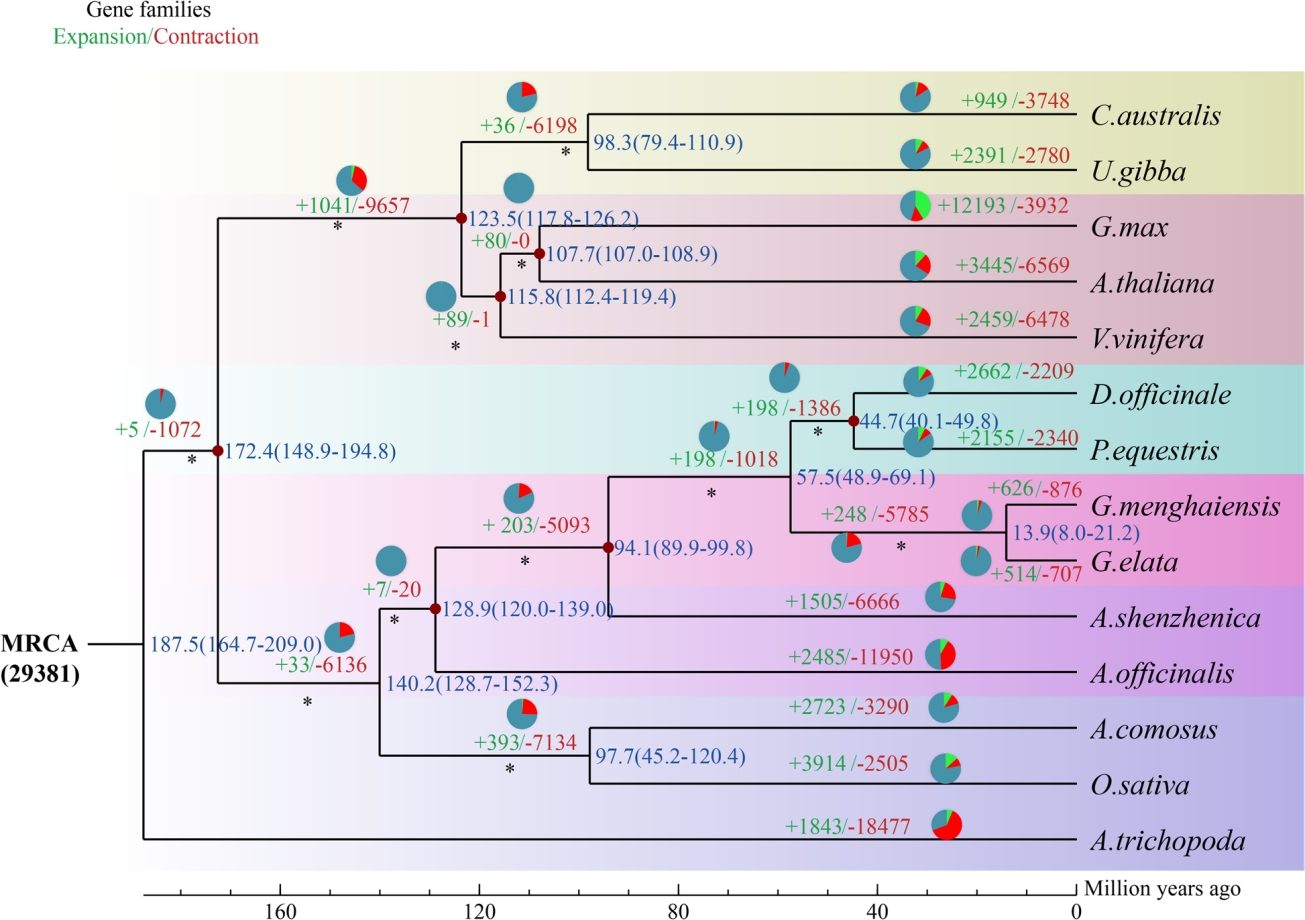
Phylogenetic position and gene families of *G. menghaiensis*. a, Inferred phylogenetic tree with 254 single-copy genes of 14 plant species. Gene family expansions are indicated in green, and gene family contractions are indicated in red. Expansions of Gene families are indicated in green, contractions of gene families are indicated in purple. Estimated divergence times (in millions of years) are indicated by light blue boxes, the red star represents the divergence time between *Gastrodia*. MRCA, most recent common ancestor

**Fig. 3:**
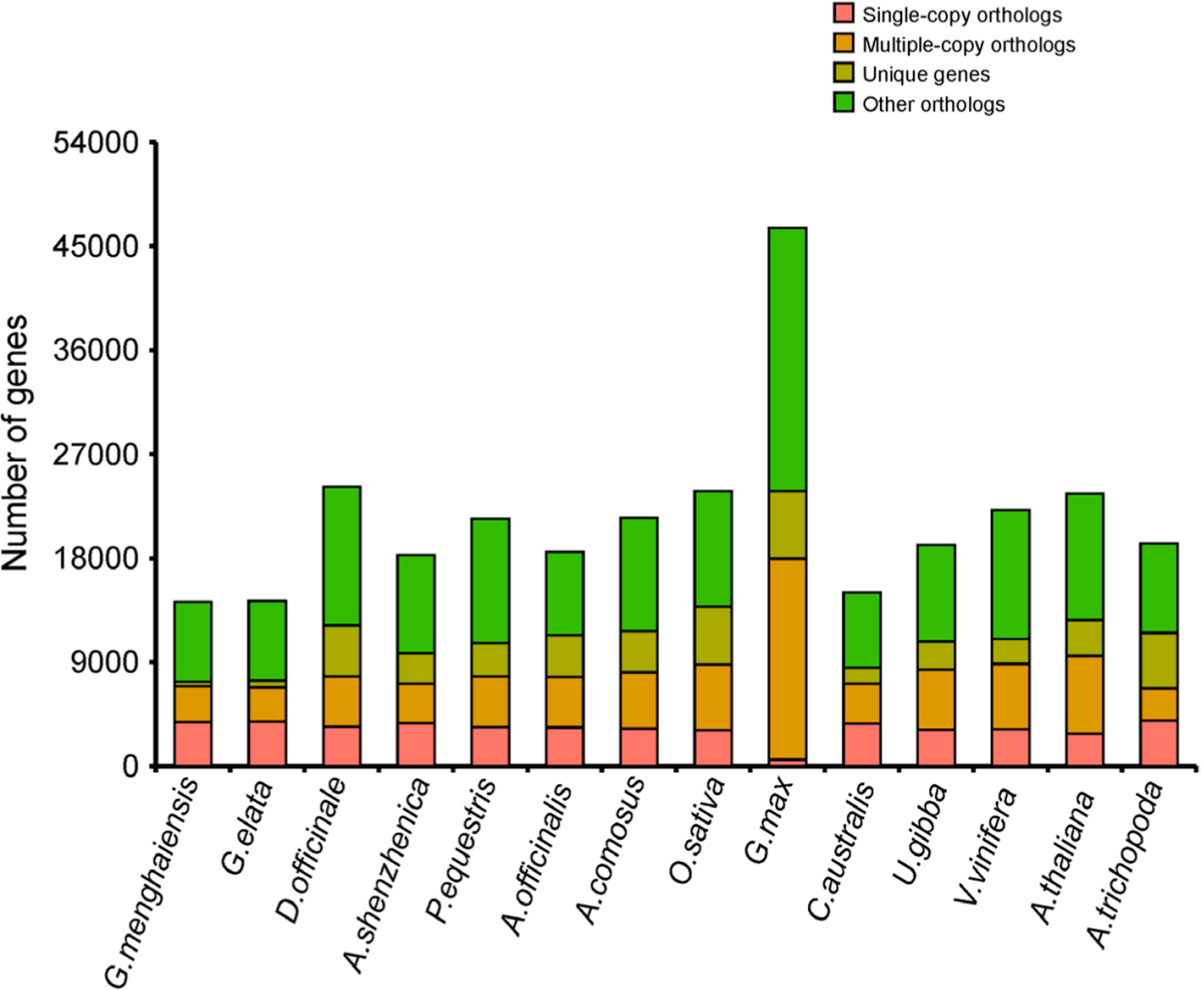
Bar graph of the number of protein-coding genes in each of 14 species. Single-copy orthologs, common orthologs with one copy in specific species; multi-copy orthologs, common orthologs with multiple copy numbers in specific species; unique gene, genes belonging to only one specific species; other orthologs, genes from families shared in 2–13 species

The divergence of *Gastrodia* from *D. officinale*/*P. equestris* was estimated at ~ 57.5 million years, and that of *Gastrodia menghaiensis* from *G. elata* at ~ 13.9 million years ago (Fig. 2). A total of 14,233 *G. menghaiensis* genes (79%) were clustered into four groups, including single-copy, multiple-copy, unique and other orthologs, containing 3,827, 3,100, 379, and 6,927 genes, respectively (Fig. 3). Among the 14 angiosperm species used in the phylogenetic analysis, *G. menghaiensis* had the smallest number of gene families and on average fewer genes in these families (Fig. 2, and Supplementary Table S13). Of 8,139 gene families shared by these five orchid species, 5,785 had decreased in *Gastrodia,* whereas 248 gene families had expanded (Fig. 2). KEGG (Kyoto Encyclopedia of Genes and Genomes) [31, 32] enrichment (FDR < 0.05) of expanded gene families of *G. menghaiensis* include tyrosine metabolism, steroid hormone biosynthesis, prolactin signal pathway, and endocytosis (Supplementary Figure S4); and contracted gene families include vitamin digestion and absorption, prolactin signal pathway, plant-pathogen interactions etc. (Supplementary Figure S5).

Compared to green orchids with 854–1,182 unique gene families, *G. menghaiensis* has 286 unique gene families (Fig. 2, Supplementary Figure S6). GO enrichment (FDR < 0.05) showed that the unique genes were mainly enriched in regulation of cyclin-dependent protein serine/threonine kinase activity, potassium channel activity, potassium ion transmembrane transport, nutrient reservoir activity (Supplementary Figure S7; Supplementary Tables S17 and S18). From most recent common ancestor of *Gastrodia* (MRCAG), 876 gene families contracted and 626 gene families expanded in *G. menghaiensis*, and 707 gene families had contracted and 516 gene families expanded in *G. elat*a (Fig. 2). Compared to *G. elata*, genes related to regulation of autophagy and nitrogen compound transport increased in *G. menghaiensis* (Supplementary Tables S18, S19 and S20). Compared to *P. equestris* [33] (29,334 PCGs), *D. officinale* (29,099 PCGs), *G. elata* [9, 29] (18,950–21, 115PCGs), and *A. shenzhenica* [34] (21,676 PCGs), *G. menghaiensis* has a relatively small proteome (17,948 PCGs), making it the smallest proteome thus far among angiosperms (Supplementary Table S13).

Among the eight species KEGG [31] annotation 132 map results, *G. menghaiensis* had significant contraction in 69 KEGG maps (the other three orchids, *C. australis*, *O. sativa*, *A. thaliana* and the two *Gastrodia* species), such as anthocyanin biosynthesis, limonene and pinene degradation, photosynthesis and pyrimidine metabolism, etc. (Supplementary Tables S21 and S22). Notably, compared with *A. shenzhenica*, *P. equestris*, *D. officinale*, the *G. menghaiensis* genome lost approximately 1073, 2590 and 2794 genes, respectively (Supplementary Table S23).

The rooting pattern of *G. menghaiensis* is characterized by well-developed branched lateral roots extending along the soil surface in the tropical forests in which it grows. We found there are 410 genes involved in root development in *G. menghaiensis*, which is similar to 411 of *A. shenzhenica*, 417 of *D. officinale*, and 429 genes for *P*. *equestris* (Supplementary Table S24). Many genes involved in adventitious root development are more numerous in *Gastrodia menghaiensis*, such as *RPT2b* [35], *MKK6* [36], *PLGG1 *[37] (Supplementary Table S24). Some genes involved in root development, such as *UTR7* (lateral root emergence) [38], *RSL2* (required for root-hair growth) [39], and *SIEL* (involved in root patterning) [40], were found in *G. menghaiensis*.

The petals and sepals of *Gastrodia* are united into a floral tube, which is different from most orchids [20]. We found that genes involved in boundaries of organs, such as *AS2*, *TP3*, *LOB1*, *LOF2*, *LBD1*, are fewer or absent in *Gastrodia* compared to the orchids with free sepals and petals (Supplementary Table S25)*.* The petal lobes are small in size with almost dorsoventral symmetry in *Gastrodia*, which is different from most orchids. We found that genes involved dorsoventral asymmetry of petals and sepals, such as *DICH* [41], *CYC* [42, 43], *RAD* [44], are fewer or absent in *Gastrodia* (Supplementary Table S25).

### The loss and relative expansion of nuclear genome copies of genes that function in plastids

**Fig. 4:**
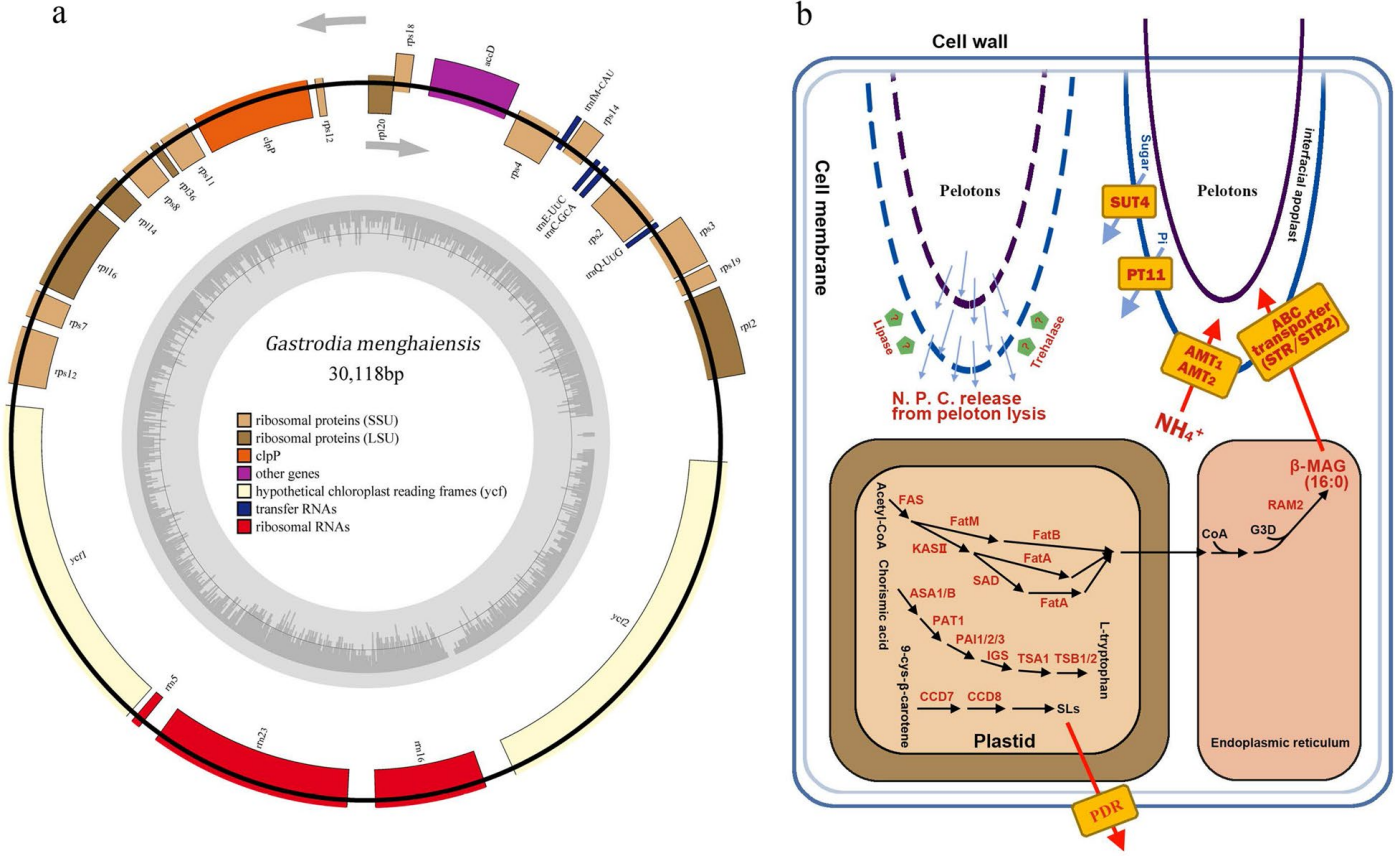
Plastid genome of *G. menghaiensis* and proposed model of biological interaction between *G. menghaiensis* and symbiotic fungi. **a** The plastid genomes of *G. menghaiensi*s. SSU, small subunit; LSU, large subunit. **b** Model of biological interaction between *G. menghaiensis* and symbiotic microbials. *ASA1/B*, anthranilate synthase; *PAT1*, phosphoribosyl tranferase; P*AI1/2/3,* PRA isomerase; *IGS*, InGP synthase; *TSA1*, Trytophan synthase; TSB1/2, Trytophan synthase; SLs, Strigolactone; *FAS*, fatty acid synthase; *KASII*, ketoacyl-ACP synthase II; SAD, stearoyl-ACP desaturase; *FatA*, acyl-ACP thioesterase A; *FatB*, acyl-ACP thioesterase B; *FatC*, acyl-ACP thioesterase C; *FatM*, acyl-ACP thioesterase M; ABC transporter, ATP binding cassette transporter; *CoA*, coenzyme A; MAG, monoacylglycerol; *SUT4*, sugar transporter 4; *PT1*1/PT4, Phosphorus transporter 11; *AMT1*, *AMT4*, ammonium transporters 1 and 4; *CCD 7*, *CCD 8*, carotenoid cleavage dioxygenases; PDR, ATP binding cassette transporter. The schematic diagrams of strigolactone, monoacylglycerol and L-trytophan pathway were edited according to KEGG and reported references [9, 46, 47]

All species of *Gastrodia* are leafless [20], so we specifically searched the *G. menghaiensis* genome for genes that mediate leaf development and found that a number of these are absent in the *G. menghaiensis* genome (Supplementary Table S24, Supplementary Figure S8). To better understand the putative functions of missing genes, we examined nuclear genes of the photosynthesis apparatus, especially chlorophyll, photosystem I, photosystem II, cytochrome b6f, cytochrome C6m, ATP synthase, and rubisco. Our results showed that that chlorophyll a oxygenase required for the chlorophyll b synthesis, together with chlorophyll degradation genes, were absent (Supplementary Tables S26 and S27)). Of the 31 nuclear genes for photosynthetic apparatus proteins (NEP), none was present in the *G. menghaiensis* genome. The plastid genome of *G. menghaiensis* (30,118 bp) was dramatically reduced in size (Fig. 4a) compared to the plastid genomes of photosynthetic orchids (see [45])( (Supplementary Table S28, Supplementary Figures S9). Most plastid genes involved in photosynthesis were lost in a manner similar to its counterpart in nuclear genome.

We found that there are approximately 696 nuclear-encoded plastid genes (NPGs) in the *G. menghaiensis* genome (Supplementary Table S26). Genes related to plastid biosynthesis of aromatic and branched amino acids and fatty acids are intact (Supplementary Tables S26 and S27). Compared to the other orchids, 28 and 38 NPGs had expanded in the genomes of *G. menghaiensis* and *G. elata*, respectively. These genes are enriched for GO terms *G. menghaiensis* in related to following metabolic processes/molecular functions (Supplementary Tables S26 and S27): (1) biosynthesis of aromatic amino acid L-tryptophan (*CM1*, *PAT1*); (2) amino acid transmembrane transport (*CAT6*); (3) starch biosynthetic process (*SS4*); (4) defense response to oomycetes (*APK2*, *CLT2*); (5) transmembrane transporter (*SAMC1*, *NDT1*); (6) monoterpenoid biosynthetic process (*TPS10*); (7) plastid ribosomal large subunit (*RPL10*, *RPL11*, *RPL28*); (8) lipase that hydrolyzes phosphatidylcholine, glycolipids as well as triacylglycerols (*DALL1*); (9) pentatricopeptide repeat (PPR) proteins for RNA editing (PCMP-E95 or AEF1); (10) plastidal glycolate/glycerate translocator 1 (*PLGG1*). For *G. elata*, NPGs enriched for GO terms include (Supplementary Tables S26 and S27): (1) branched chain amino acid (*AL*S); (2) biosynthesis of aromatic amino acid L-tryptophan (*ASB*, *PAT1*); (3) fatty acid biosynthetic process (*accD*); (4) lipase that hydrolyzes phosphatidylcholine, glycolipids as well as triacylglycerols (*DALL1*); (5) transmembrane transporter (*SAMC1, DIT1*); (6) plastid ribosomal subunits (*RPL12*, *RPL14*, *RPL2-A*, *RPL9*, *RPS11*, *RPS12*, *RPS3*); (7) strigolactone biosynthetic process (*CCD7*); (8) starch biosynthetic process (*SBE3*); (9) starch degradation (*R1*); (10) plastid division protein (*CDP1*).

### Biological interaction between *G. menghaiensis* and symbiotic microbionts

Mycorrhizal symbioses have been important mutualistic associations between plants and soil fungi for 460 million years, and this link is likely an ancestral feature of all terrestrial plants [48, 49]. Plants depend on soil fungi for uptake of minerals and water, and fungi obtain essential nutrients (carbohydrates and amino acids) from their partners [5, 49–51]. It is hypothesized that mycorrhizal symbioses have triggered the contemporaneous radiations of fungi and plants [48, 49]**. S**ome green orchids obtain organic carbon from both photosynthesis and their mycorrhizal fungi [8, 18]**.** With total loss of photosynthesis function, leafless *G. menghaiensis* fully depends on its mycorrhizal partners for organic carbon throughout its life cycle. Mycoheterotrophic plants are an ideal model system to illustrated plant–fungus interactions.

In general for photosynthetic orchids, fungi provide amino acids to the orchids in exchange for minerals and water [52]. It has been hypothesized that interactions between some orchids and their symbiotic microbionts are similar to those between other plants and arbuscular mycorrhizae (AM) [18, 53]. Genes related to colonization of AM were found in five orchids specie genomes, including the development of AM symbiosis (*EXO70*, SNARE family) [54, 55], fatty acid biosynthesis in plastid and endoplasmic reticulum (*FatM*, *KASI*, *FAS*, *RAM1*, *RAM2*) [53, 56, 57], fatty acid transporter (*STR/STR2*) [57], ammonium transporters (*AMT1, AMT2*) [58, 59], phosphate transporter (*PT11-PT4*) [60] (Fig. 4b; Supplementary Table S29), and sugar transporter (*SUT4*) [14] (Supplementary Table S30). Transcriptome data indicated that most genes were expressed or highly expressed in roots of *G. menghaiensis* (Supplementary Figures S10 and S11)*.* Suetsugu et al. (2017) indicated that fully mycoheterotrophic albino individuals of *Epipactis helleborine* (Orchidaceae) had upregulated expression of genes related to AM [25]. The loss of NTR (nitrate transporters) and expansion of the number of genes for urease in *Gastrodia* indicated that its uptake of nitrogen is mainly in form of ammonium (Supplementary Table S30). *SUT4* has been revealed to mediate the transport of sugar from mycorrhizal fungi to *G. elata *[14]*.*

The interaction with mycorrhizae is crucial for survival of *G. menghaiensis*. LysM receptor-like kinases (LysM-RLK) mediate this process with AM fungi in plants [61]. Four LysM-RLK were found in the *G. menghaiensis* genome (Supplementary Table S31). Transcriptome data indicated that two of them were highly expressed in roots (Supplementary Figure S12). It is well known that strigolactones, a class of plant hormones, stimulate AM fungal pre-symbiotic growth [62, 63]. Specifically, strigolactone can stimulate hyphal branching and development of symbiotic fungus *A. mellea* in *G. elata* [9]. Key genes for biosynthesis and secretion of strigolactone (*CCDS*, *PDKs*) were expanded in *G. menghaiensis* genome (Supplementary Table S32).

There are lots of debates about the way in which carbon transferred from fungi to orchids [64–66]. Trehalose is an abundant fungal soluble carbohydrate [67]. Smith (1967) suggested that trehalose moved from fungi hyphae to orchid cells as carbon nutrients [65]. Ponert et al. (2021) indicated that orchid protocorms possess an efficient and trehalase-dependent pathway for utilizing exogenous trehalose [64]. Expansion of genes encoding trehalase in genomes of *Gastrodia* indicated that *G. menghaiensis* might have developed the ability to use trehalose as its organic carbon source. The pelotons are highly dynamic, and degradation of pelotons also releases large amounts of organic carbon and nitrogen to orchids [68]. Glucans and chitin are two main components of fungal cell walls [69, 70]. There are 36 beta-glucosidase genes (Supplementary Table S29) and four glycoside hydrolase family 18 (GH18) chitinases (Supplementary Table S30). These genes may be involved in the degradation of the cell wall of fungi to provide nutrients for *G. menghaiensis*, although extensive degradation of fungal tissues in orchids is not typical.

Hyphae of orchid mycorrhizal fungi usually form pelotons in root cells of orchids. The rooting pattern of *G. menghaiensis* is characterized by well-developed branched lateral roots extending along the soil surface in the tropical forests in which it grows (Figure S1a). We found there are 410 genes involved in root development in *G. menghaiensis*, which is similar to the 411 of *A. shenzhenica*, 417 of *D. officinale*, and 429 genes for *P*. *equestris* (Supplementary Table S24). Many genes involved in adventitious root development are more numerous in *Gastrodia menghaiensis*, such as *RPT2b *[35], *MKK6* [36], *PLGG1 *[37] (Supplementary Table S24). Some genes involved in root development, such as *UTR7* (lateral root emergence) [38], *RSL2* (required for root-hair growth) [39], and *SIEL* (involved in root patterning) [40], were found in *G. menghaiensis* but absent in the other orchids including *G. elata*. In particular, two genes related to lateral root development, *ASL18a* and *NF-Y *[71, 72], are present in the *G. menghaiensis* genome but absent from the other orchid genomes. *ASL18a* and *NF-Y* together regulate nodule organogenesis in legumes [71, 72]. Although *G. elata* has lost roots, there are 424 genes involved in root development in *G. elata*. However, many genes essential for root development, such as *PSP*, *VPS26C*, *PI-4KBETA2* and *PI-4KBETA1*, were lost in *G. elata* (Supplementary Table S24).

Although *G. menghaiensis* depends on mycorrhizal fungi for life, it still requires protection against attack by pathogens and thus retains defense-related genes. The *G. menghaiensis* genome contains 28 terpene synthase genes, which defend against pathogens [73, 74], but there are 15, 29 and 43 *TPSs* in the other orchids (Supplementary Table S33). The *G. menghaiensis* genome contains 65 *R* genes *R* (resistance), which are important components of plant defense system, which is similar to the number in *A. shenzhenica* but fewer than the other two autotrophic orchid species. The *G. menghaiensis* genome contains 145 *P450s*, whereas there are 123 *P450s* in the genome *A. shenzhenica* (Supplementary Table S33). Compared to the other orchids, 143 genes involved in plant resistance to pathogens, such as *DIR15*, *SBT3.3*, *TL1* [39], are increased in the *G. menghaiensis* genome (Supplementary Table S34).

## Materials and methods

### Genome sequencing

The *Gastrodia menghaiensis* used for sequencing was collected from Mengsong, Menghai County, Yunnan Province, China. We had permission from local Forest Department to collect plants for this study. Healthy flowering plants were collected and washed three times with ultrapure water. Then, the plants were immediately frozen in liquid nitrogen and stored at -80 °C prior to DNA extraction. Total DNA was extracted from inflorescences of *G. menghaiensis* [26, 75] (removing corms and roots) with the DNAsecure Plant Kit (TIANGEN) and cut into random fragments.

We constructed the DNA sequencing libraries and paired-end library with insert size of 350 bp following the standard Illumina library preparation protocols and the manufacturer’s instructions (Illumina, San Diego, CA), respectively. Short-read libraries were sequenced on Illumina HiSeq 2500. We filtered out adapter sequences and the low-quality and duplicated reads, a total of 122.51 Gb of data remained for the assembly.

For Pacbio libraries, at least 10 μg of sheared DNA was required. The SMRT bell template preparation involved DNA concentration, damage repair, end repair, ligation of hairpin adapters, and template purification. SMRT Bell libraries with an insert size of 40 kb were constructed and then sequenced on the PacBio Sequel platform (Pacific Biosciences, USA) using the P6 polymerase/C4 chemistry combination, based on the manufacturer’s procedure (Pacific Biosciences, CA, USA). A total of 100.48 Gb of (102.7-fold coverage of whole genome) data were retained (Supplementary Table S2).

For 10X Genomics libraries, approximately 1 ng of input DNA with 50 kb length was used for the GEM reaction procedure during PCR, and 16-bp barcodes were introduced into droplets. The plant cells (removed the corms and roots) were lysed and HindIII endonuclease was used for digesting the fixed chromatin. The 5’ overhangs of the DNA were recovered with biotin-labeled nucleotides and the resulting blunt ends were ligated to each other using DNA ligase. Proteins were removed with protease to release the DNA molecules from the crosslinks. Then, the droplets were fractured following the purification of the intermediate DNA library. The libraries were finally sequenced on the Illumina Hiseq 2500. Finally, a total of 129.04 Gb (131.9-fold coverage of the genome) data were retained (Supplementary Table S2).

### Genome assembly

We estimated the genome size of *G. menghaiensis* by analyzing the K-mer frequency. Based on 122.59 Gb pair-end reads (350 bp) and the k-mer analysis, we found that the distribution of the17-mer depends on the characteristics of the genome and follows a Poisson’s distribution. The *G. menghaiensis* genome size was estimated about 988.74 Mb (Supplementary Table S1, Supplementary Figure S2).

De novo assembly of the long reads from the PacBio SMRT Sequencer was performed using FALCON (https://github.com/PacificBiosciences/FALCON/) [76]. To obtain enough corrected reads, the longest coverage of subreads was first selected as seed reads to correct sequence errors. Then, error-corrected reads were aligned to each other and assembled into genomic contigs using FALCON with the following parameters: length_cutoff_pr = 10,000, max_diff = 95, and max_cov = 105. Then, genomic contigs were polished using Quiver [77], which yielded an assembly with a contig N50 size of 2.37 Mb. The total length of this assembly version was 859.11 Mb. Then, we used BWA-MEM [78] to align the 10X Genomics data to the assembly using default settings. Scaffolding was performed by FragScaff [79] with the barcoded sequencing reads. Last, Pilon [80] was used to perform error correction based on the Illumina sequences, generating a genome with a scaffold N50 size of 6.82 Mb. The total length of this assembly version was 862.84 Mb. Subsequently, the Hi-C sequencing data were aligned to the assembled scaffolds by BWA-mem [78] and the scaffolds were clustered onto chromosomes with LACHESIS [81] (http://shendurelab.github.io/LACHESIS/), the final genome was 862.86 Mb and the contig and scaffold N50 were 2.02 Mb and 54.12 Mb, respectively (Supplementary Table S3).

### RNA sequencing and assembly

Five tissues of *G. menghaiensis*, including flower buds, blooming flowers, stems, young fruits, roots, were collected from Menghai County, Yunnan Province, China. All collected samples were washed with ultrapure water then immediately kept in RNALater and stored at -80 °C prior to RNA extraction. For each tissue, three biological replicate samples were analyzed. The total RNA was extracted from all samples using genomic DNA contamination and removed using RNase-Free DNase I (TIANGEN). The integrity of RNA was evaluated on a 1.0% agarose gel stained with ethidium bromide (EB), and its quality and quantity were assessed using an Agilent 2100 Bioanalyzer (Agilent Technologies). The cDNA library was constructed using the NEBNext Ultra RNA Library Prep Kit for Illumina, following the manufacturer’s recommendations. Library preparations were sequenced on an Illumina Hiseq 2500 platform, generating 150 bp paired-end reads.

Clean data were obtained by removing reads containing adapter, reads containing ploy-N and low-quality reads from raw data. We mapped clean reads and high-quality reads to the draft reference genomes by TopHat2 [82] with following the parameters: –max-intron-length 500,000, –read-gap-length 10, –read-edit-dist 15, –max-insertion-length 5 and –max-deletion-length 5. RPKM value for each protein-coding gene was calculated by HTSeq [83] using default parameters. DESeq2 [84] were used for nomorlizing gene expression (BaseMean) in each sample, and identified differentially expressed genes (DEGs) for each compared group by using P-adj (adjusted *p* value) < 0.05 as the threshold. GO enrichment analysis of DEGs was implemented by the GOseq R package [85], in which the gene length bias was corrected. GO terms with corrected *P*-values less than 0.05 were considered significantly enriched by DEGs. We used KOBASsoftware [31] to test the statistical enrichment of DEGs in KEGG pathways. Pathways with q-values < 0.05 were considered significantly enriched.

### Genome annotation

To predict protein-coding genes in the *G. menghaiensis* genome, we used homology-based prediction, de novo prediction and transcriptome prediction. Homolog proteins from six plant genomes, *Gastrodia elata* [9], *Dendrobium officinale* [86], *Apostasia shenzhenica* [34], *Phalaenopsis equestris* [33], *Oryza sativa* [87], *Ananas comosus* [88], were downloaded from Ensemble Plants (http://plants.ensembl.org/index.html, ensembl.plant.v32).

Protein sequences from these genomes the aligned to the *G. menghaiense* genome assembly using TblastN [89] with an E-value cutoff of 1e^−5^. The BLAST hits were conjoined by Solarsoftware [90], and low-quality records were removed. GeneWise [91] was used to predict the exact gene structure of the corresponding regions for each BLAST hit (Homo-set). For transcriptome-based prediction methods, RNA based prediction methods, RNA-seq data seq data were mapped to the assembly using Tophat (version 2.0.13) [82] and Cufflinks [92] (version 2.1.1), and then the transcripts were assembled into gene models (Cufflinks-set). In addition, RNA-seq data were assembled by Trinity [93] (r20140413p1), creating pseudo-ESTs and ESTs. These pseudo-ESTs were also mapped to the assembly, and gene models were predicted by PASA [94]. This gene set was denoted PASA-T-set (PASA Trinity set) and was used to train ab initio gene prediction programs. Five ab initio gene prediction programs, Augustus (version 2.5.5) [95], Genscan (version 1.0) [96], GlimmerHMM (version 3.0.1) [97], Geneid (version 1.4) (23) [98] and SNAP [99] (version 2006–07-28) were used to predict coding regions in the repeat-masked genome. Gene model evidence from the Homo–set, Cufflinks–set, PASAT–set and ab initio programs set and ab initio programs was combined by EvidenceModeler (EVM) [100] into a non-redundant set of gene structures. Finally, a total of 17,948 genes were predicted genes were predicted from the *G. menghaiensis* genome (Supplementary Table S13).

The functional annotation of the protein-coding genes was achieved using BLASTP (version 2.2.28) (with an E-cutoff of 1e^−5^) against two integrated protein sequence databases: SwissProt (https://web.expasy.org/docs/swis--protprot/guideline.html) and NR (version 20,190,709). Protein domains were annotated by searching against the InterPro (version 32.0) [101] and Pfam (version 3.0) databases using InterProScan (version 4.8) and HMMER [102] (version 3.1b1), respectively. The Gene Ontology (GO) terms for each gene were obtained from the corresponding InterPro or Pfam entry. The pathways in which the genes might be involved were assigned by BLAST against the KEGG databases (release 20,190,601) with an E-value cutoff 1e^−5^. We used the same method to re-annotate six reference genomes (*Dendrobium officinale*, *Apostasia shenzhenica*, *Phalaenopsis equestris*, *Asparagus officinalis*, *Oryza sativa*, *Arabidopsis thaliana*). A total of 15,152 genes were predicted to be functional, accounting for 84.42% of all genes in the *G. menghaiensis* genome (Supplementary Table S15). Annotation features such as the distributions of mRNA length, exon length, exon number, intron length and CDS length are shown in (Supplementary Figure S2, Supplementary Table S14). Gene structures were predicted with a combination of homology-based prediction, de novo prediction and transcriptome-based prediction. We then generated functional assignments of the *G. menghaiensis* genes with BLAST in public protein databases, including SwissProt (https://web.expasy.org/docs/swis--protprot/guideline.html), NR (version 20,190,709), Protein domains were annotated by searching against the InterPro (version 32.0), Pfam (version 3.0) and KEGG (release 20,190,601)(https://www.kegg.jp/).

A total of 62.57% repeat sequences in the genome were annotated. Among them, TEs were identified by combining de novo-based and homology-based approaches using RepeatModeler (version 1.0.4) (http://www.repeatmasker.org/RepeatModeler/ RepeatModeler/), LTR_FINDER (version 1.07) (http://tlife.fudan.edu.cn/ltr_finder/ finder/), RepeatScout (version 1.0.5) (http://www.repeat masker.org/) and Piler (version 1.0) (http://www.drive5.com/piler/), RepeatMasker (version 3.3.0) (http://www.repeatmasker.org/org/) and RepeatProteinMask (http://www.repeatmasker.org/org/). Tandem repeats were detected using Tandem Repeats Finder (TRF) (Supplementary Table S12).

Noncoding RNA was predicted using de novo and homology search methods. The tRNA genes were identified by tRNAscanSE software [103] (version 1.3.1). The rRNA fragments were predicted by aligning to the rRNA sequences using BlastN with an E-value value cutoff 1e^−10^. The miRNA and snRNA genes were predicted by INFERNAL softwares (version 1.1) [104] against the Rfam database (release 11.0) [105]. Finally, we predicted the transfer RNA genes, miRNA genes, small nuclear RNA genes, and ribosomal RNA genes in the *G. menghaiensis* genome (Supplementary Table S16).

### Quality evaluation for genome assembly

We evaluated draft assembly by mapping the high-quality reads from short insert-size PE libraries to the scaffolds using BWA-mem [106]. The distribution of the sequencing depth at each position was calculated using SAMtools to assess the completeness of the genome assembly. Approximately 97.66% of the reads could be mapped to the assembly, which covered 99.55% of the assembled sequence (Supplementary Table S4).

To assess the quality of the genome assembly, we assembled the transcriptome data of *G. menghaiensis* using Trinity [93], and generated 100,217 unigenes. These unigenes were then mapped to the scaffolds using BLAT [107]). More than 99.63% of these unigenes could be identified in the assembly, indicating that the assembly has good coverage of the gene regions (Supplementary Table S5). The CEGMA (Core Eukaryotic Genes Mapping Approach) pipeline was used to assess the completeness of the genome assembly or annotations. Analysis of the genome assembly for core eukaryotic genes revealed homologs for 93.55% of conserved genes in the assembly (Supplementary Table S6 and S8). We also used BUSCO (Benchmarking Universal Single-Copy Orthologs) to quantitatively assess of genome assembly, gene set, and transcriptome completeness based on evolutionarily informed expectations of gene content from near-universal single-copy orthologs selected from embryophyta_odb9. We found 67.9% conserved genes in the *G. menghaiensis* genome (Supplementary Table S8).

### Gene family construction

Protein sequences from *G. menghaiensis* and the thirteen other sequenced plant genomes with representatives from *Gastrodia elata* [9], *Dendrobium officinale* [86], *Apostasia shenzhenica* [34], *Phalaenopsis equestris* [33], *Asparagus officinalis* [108], *Oryza sativa* [87], *Ananas comosus* [88], *Cuscuta australis* [109], *Utricularia gibba* [110], *Vitis vinifera* [111], *Glycine max* [112], *Arabidopsis thaliana* [113] and *Amborella trichopoda* [114] were used for gene family clustering. These 14 species include the angiosperm sister to all others, five core dicots, and seven monocots. Four out of the five orchids with sequenced genome were studied. *Arabidopsis thaliana* [113], *Glycine max*[112], *Oryza sativa*, are model plants; *Asparagus officinalis* and the orchids belong to same monocot order, Asparagales. All 14 species protein datasets were clustered into paralogous and orthologous using the program OrthoMCL (http://orthomcl.org/orthomcl/) with the inflation parameter 1.5.

### Phylogenetic tree and divergence estimation

We aligned all 254 single-copy gene protein sequences by MUSCLE (http://www.drive5.com/muscle/) and combined alignment results to build a super alignment matrix. Then, the phylogenetic tree of 14 species was constructed using RAxML (version 8.0.19) (http://sco.h-its.org/exelixis/web/software/raxml/index.html) with the maximum likelihood method and a bootstrap of 100. *A. trichopoda* was used as outgroup. The MCMC tree program (http://abacus.gene.ucl.ac.uk/software/paml.html) implemented in phylogenetic analysis by maximum likelihood (PAML) was applied to infer the divergence time based on the phylogenetic tree constructed. The calibration times of the divergence between *Dendrobium officinale* and *Phalaenopsis equestris*, *Apostasia shenzhenica* and other orchid species, *Oryza sativa* and *Ananas comosus*, *Glysine max* and other monocots were obtained from the TimeTree database (http://www.time.org/) and previous results [19, 115, 116].

### Expansion and contraction of gene families

We determined expansion and contraction of the gene families by comparing the cluster size differences between the ancestor and each species using the CAFÉ [117] (version 4.0). Functional categories that were enriched for significant gene family expansions mainly included terpene synthase activity, magnesium ion binding, serine-type endopeptidase activity and so on (Supplementary Table S22).

### Analysis of R genes, terpene synthase and *P450s*

To discover R genes in *G. menghaiensis* genome, we screened for the presence of NB-ARC domain (PF00931) with HMMER(version 3.1b1), resulting in a total of 65 R genes in *G. menghaiensis*. The NB-ARC domain was also identified for the 6 Ref-Species to discover the R genes in these reference species (Supplementary Table S23). We identified 28 terpene synthase (TPS) by requiring the presence of both the N-terminal domain PF01397 and C-terminal domain PF03936 [118, 119] in the *G. menghaiensis* genome. The same method was also applied to search for TPSs of the 6 Ref-Species (Supplementary Table S24). P450 genes were identified using PFAM with PF00067 using HMMER (version 3.1b1) (Supplementary Table S23).

### The assembly and analysis of plastid genome of *G. menghaiensis*

The cleaned reads approximately 5 Gb from Illumina HiSeq 2500 were used to assemble the plastid genome (plastome) of *G. menghaiensis* following methods in Li et al. [120]. The finished plastome scaffolds were reoriented according to the *C. triplicata* reference plastome. Linear plastome maps were drawn using OGDRAW.

Completed plastomes were annotated using PGA [121].

### Identification of genes involved in leaf and root development, fusion of sepals and petals and floral symmetry in *G. menghaiensis*

To discover leaf development genes in *G. menghaiensis*, the complete published list of *Arabidopsis* leaf development genes (327 genes) [122, 123] were used as the queries to blast to 6 Ref-Species to identify the candidate genes. The BLAST hits were conjoined by Solar software, then we compared the consistency of domain of the query and ref-genes use HMMER (version 3.1b1)(Supplementary Table S35). To discover root development genes in *G. menghaiensis*, the complete published list of *Arabidopsis* root development genes [113, 124–126] (540 genes) were used as the queries to blast to 6 Ref-Species to identify the candidate genes. To discover the genes involved the fusion of petals and petals and floral symmetry in *G. menghaiensis*, we used the genes mentioned in as queries to blast to 6 Ref-Species to identify the candidate genes. The BLAST hits were conjoined by Solar software, the blast result was filtered and compared the consistency of domain of the query and ref-genes using HMMER(version 3.1b1) (Supplementary Table S36).

### Cytological studies on *Gastrodia menghaiensis*

Chromosomes of *Gastrodia* spp. are often diffuse and indistinct, and the size is small [127]. Karyotype of *Gastrodia menghaiensis* was studied in 2019 and 2020 following methods of Jin et al. [128]. Briefly, fresh root tips about 0.2 cm in length were cut in field, pretreated in 0.002 M 8-hydroxyquinoline at 20 °C about 3 to 4 h. Experiments were repeated five times, we observed ten or more slides for each time. The chromosome numbers of *G. menghaiensis* are 2n = 36 (Supplementary Fig. 13).

## Supplementary Information

**Additional file 1.**

**Additional file 2.** 

## Data Availability

The genome assembly and annotation data of *Gastrodia menghaiensis* have been deposited to the Figshare database (https://figshare.com/s/f759784e78e86ba71c7c). The raw sequencing data used for de novo whole-genome assembly and the RNA-seq data for the annotation of *G. menghaiensis* have been deposited to the NCBI Sequence Read Archive (SRA) under GenBank Bioproject PRJNA695369. The biosample for raw sequencing data is SAMN17216907 and for RNA-seq data SAMN17216905. When the manuscript is accepted, all genome assembly and annotation data will be publicly available.

## Abbreviations

BUSCO: Benchmarking universal single-copy orthologs analysis
CEGMA: Conserved core eukaryotic gene mapping approach
EST: Expressed Sequence Tag
GO: Gene Ontology
KEGG: Kyoto Encyclopedia of Genes and Genomes
LTRs: Long terminal repeats
PCGs: Protein-coding genes
