# Supplementary material for: The Gastrodia menghaiensis (Orchidaceae) genome provides new insights of orchid mycorrhizal interactions
Source: BMC Plant Biol. 2022 Apr 7;22:179. doi: 10.1186/s12870-022-03573-1 (PMC8988336; doi:10.1186/s12870-022-03573-1)
Supplement: Supplementary file 1 — Additional file 1. [file 12870_2022_3573_MOESM1_ESM.docx]

**Supplementary Information**

**The *Gastrodia* (Orchidaceae) genomes provide new insights of orchid mycorrhizal interactions**

Yan Jiang^1^, Xiaodi Hu^2^, Yuan Yuan^3^, Xueliang Guo^1^, Mark W. Chase^4,6^, Song Ge^1^, Jianwu Li^5^, Jinlong Fu^2^, Kui Li^2^, Meng Hao^2^, Yiming Wang^2^, Yuannian Jiao^1^, Wenkai Jiang^2^ & Xiaohua Jin^1^

^1^Institute of Botany, Chinese Academy of Sciences, Xiangshan, Haidian, 100093 Beijing, China.

^2^Novogene Bioinformatics Institute, 100083 Beijing, China

^3^ National Resource Center for Chinese Meteria Medica, Chinese Academy of Chinese Medical Sciences, Chaoyang, 100700 Beijing, China.

^4^ Jodrell Laboratory, Royal Botanic Gardens, Kew, Richmond, Surrey TW9 3DS, UK

^5^ Xishuanbanan Tropical Botanical Gardens, Chinese Academy of Sciences, Menglun, Mengla, Yunnan, China.

^6^Department of Environment and Agriculture, Curtin University, Perth, Western Australia, Australia.


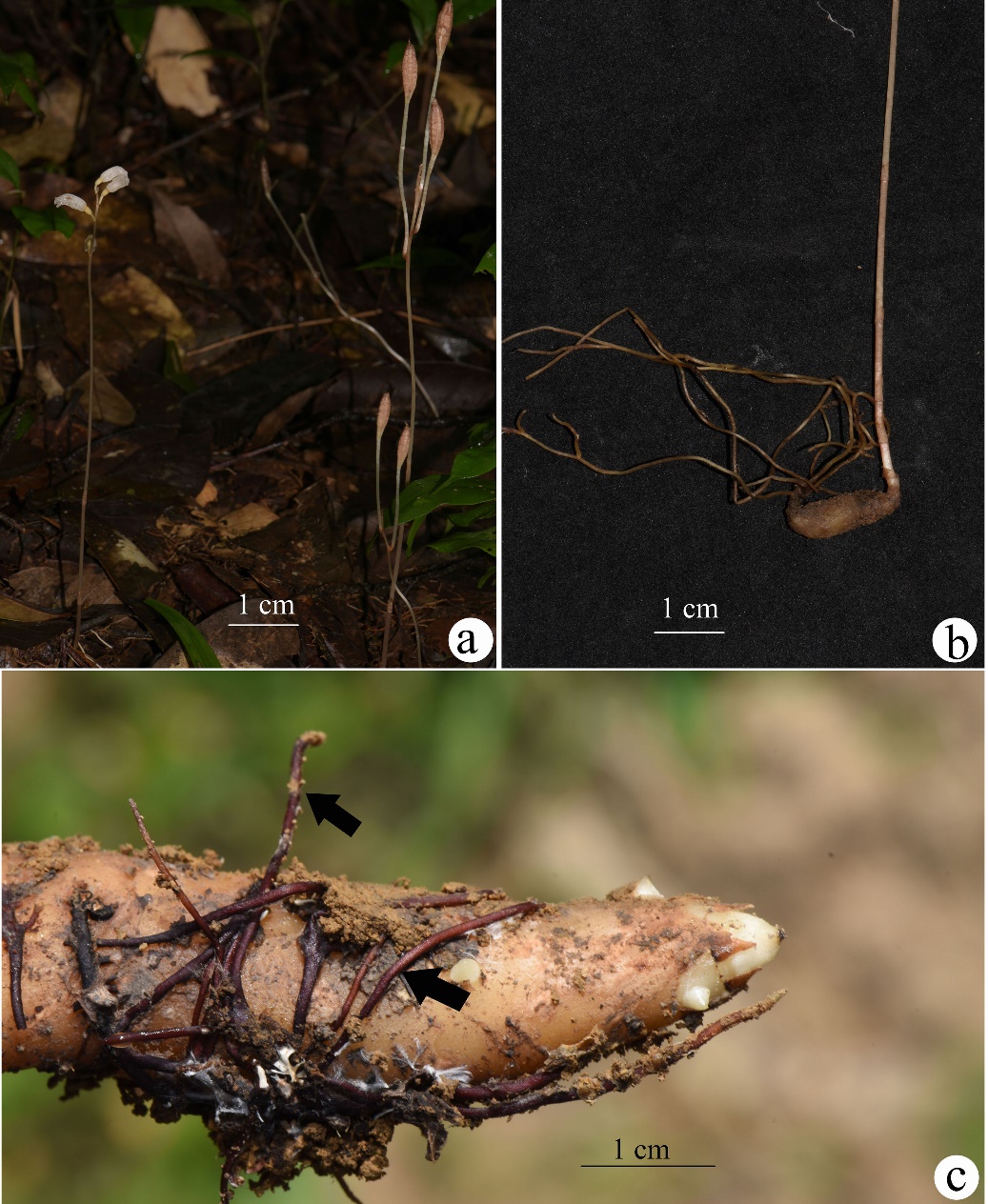


**Supplementary Figure S1. Habit of *Gastrodia menghaiensis* and *G. elata***

a, plants of *G. menghaiensis* in habitat; b. tubers and roots of flowering plant of *G. menghaiensis*; c. tuber of *G. elata*. Arrows indicating rhizomorph. d. genome characteristics of *Gastrodia menghaiensis*.


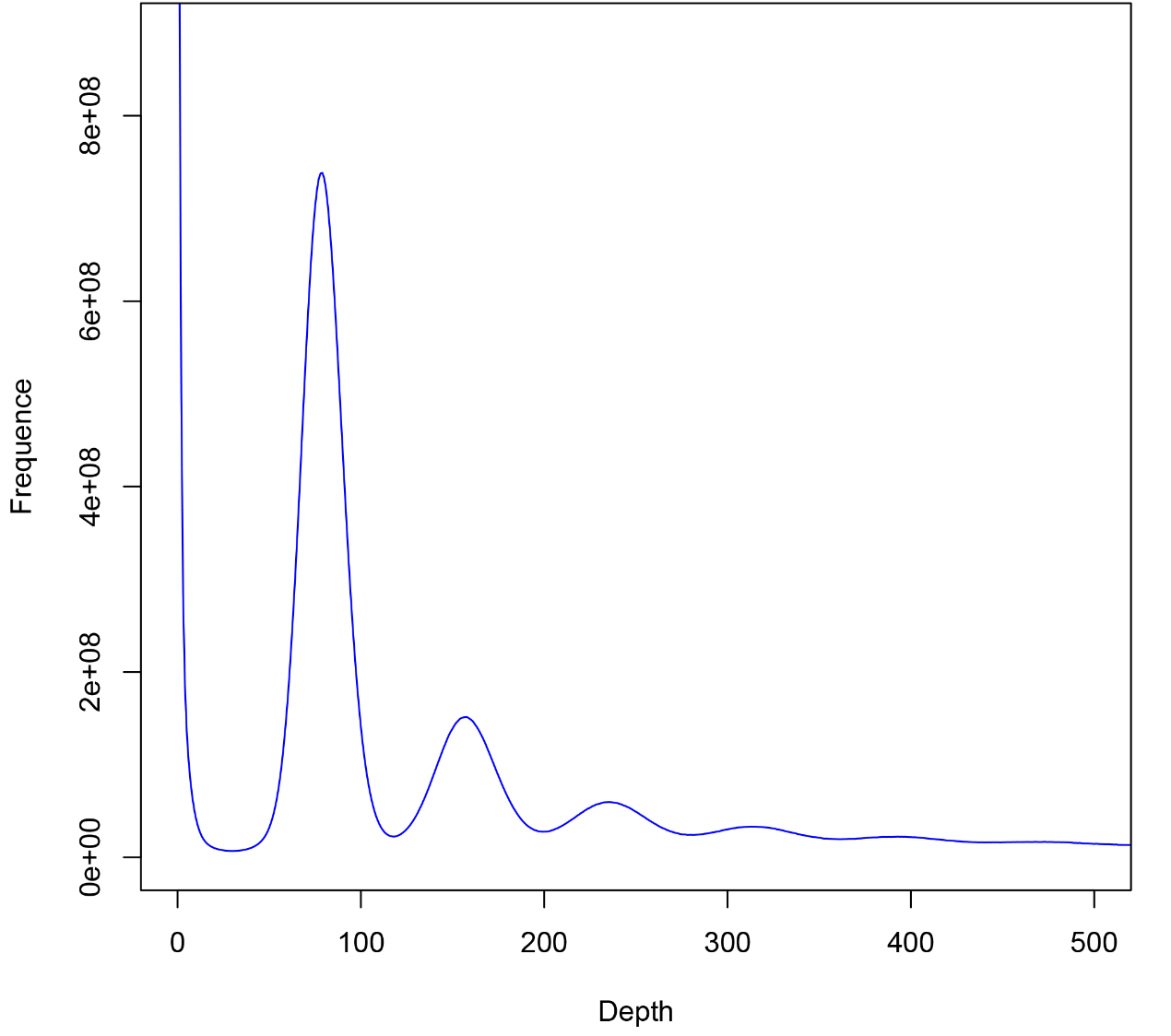
**Supplementary Figure S2. K-mer analysis for estimating the genome size of *G. menghaiensis****.* The paired-end reads from short insert-size libraries (350 bp) were used to generate the 17-mer frequency curve. The horizontal axis represented the number of times occurred. The volume of K-mers was plotted against the frequency at which they occur. The left-hand peak at low frequency and high volume represented K-mers containing essentially random sequencing errors. The main volume peak of K-mers was 105.


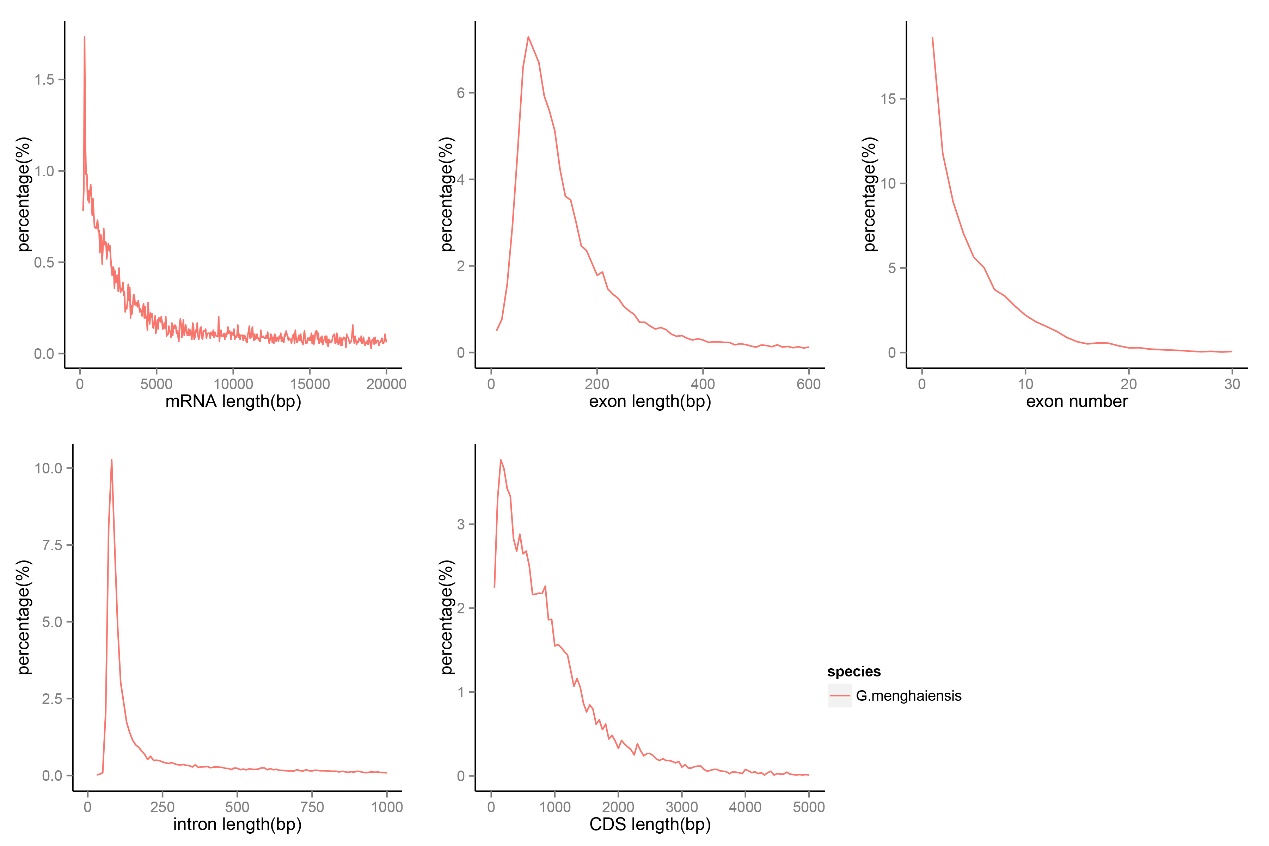


Supplementary Figure S3. Length distribution of CDSs, genes, exons, and introns of *G. menghaiensis*. X-axis, length; Y-axis, the percent of genes.


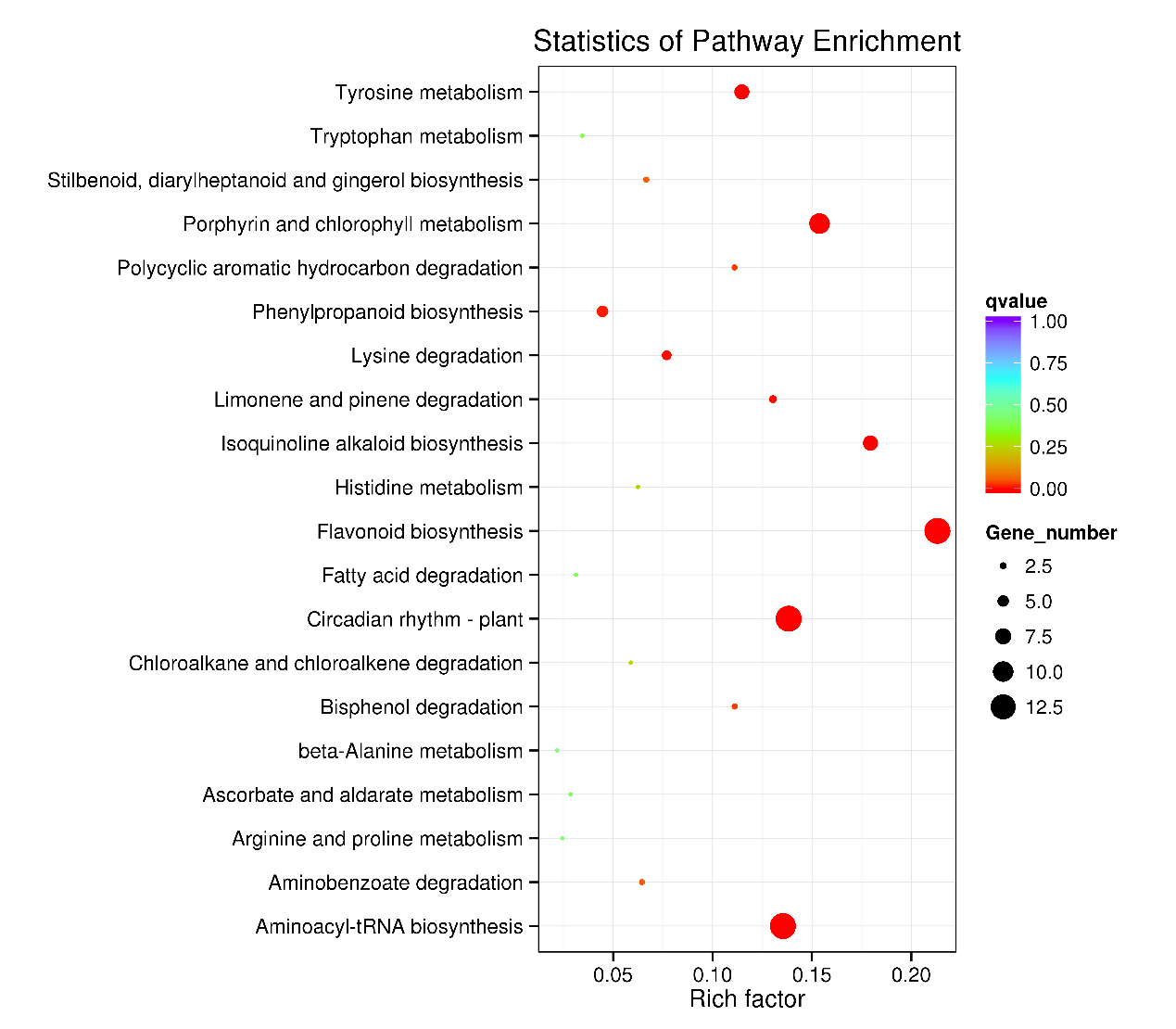


**Supplementary Figure S4. KEGG enrichment of expanded gene families of *G. menghaiensis*** (KEGG, see [www.kegg.jp/kegg/kegg1.html](http://www.kegg.jp/kegg/kegg1.html), [1, 2])**.**


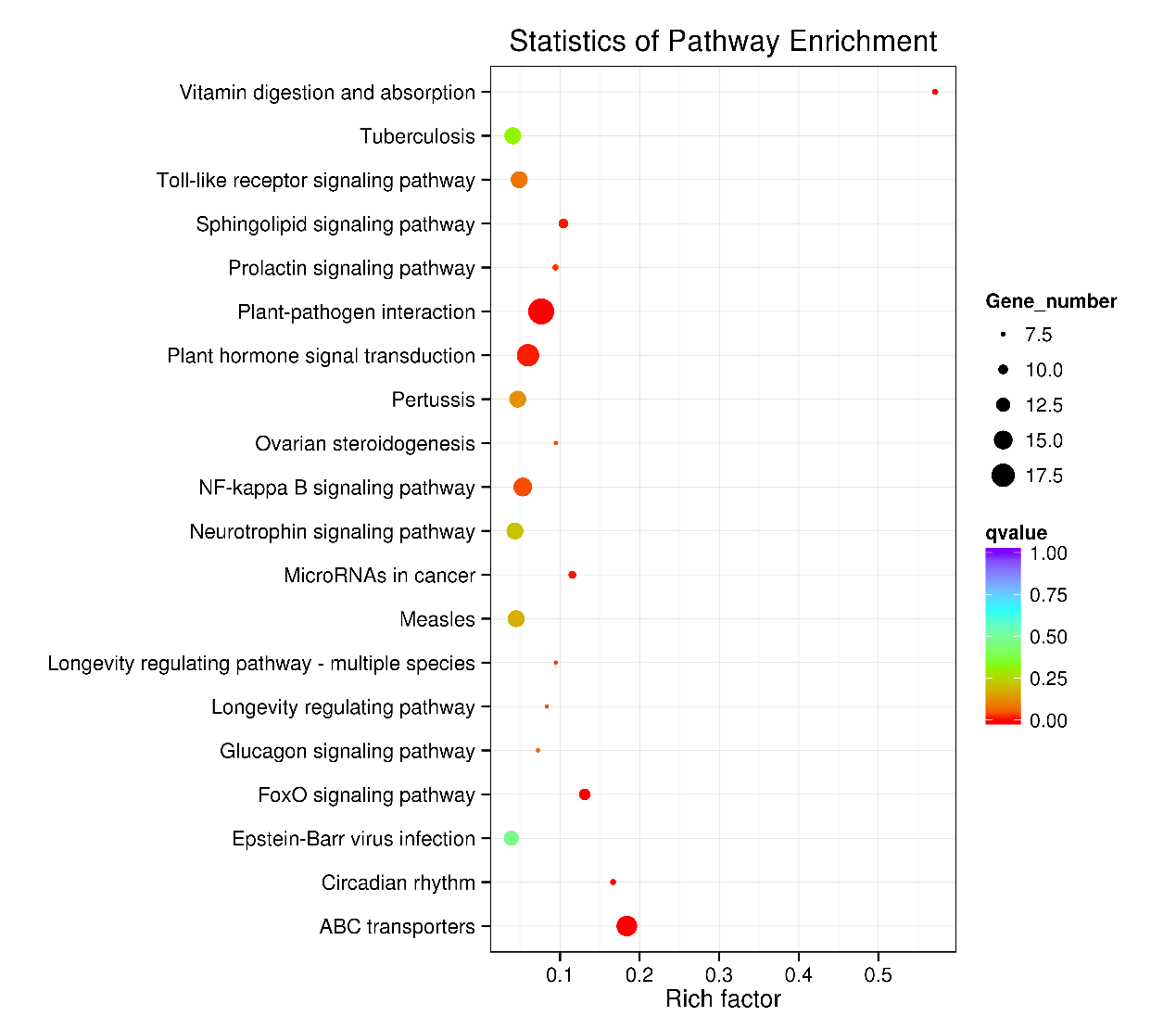


**Supplementary Figure S5. KEGG enrichment of contracted gene families in *G. menghaiensis* genome** (KEGG, see [www.kegg.jp/kegg/kegg1.html](http://www.kegg.jp/kegg/kegg1.html), [1, 2]).


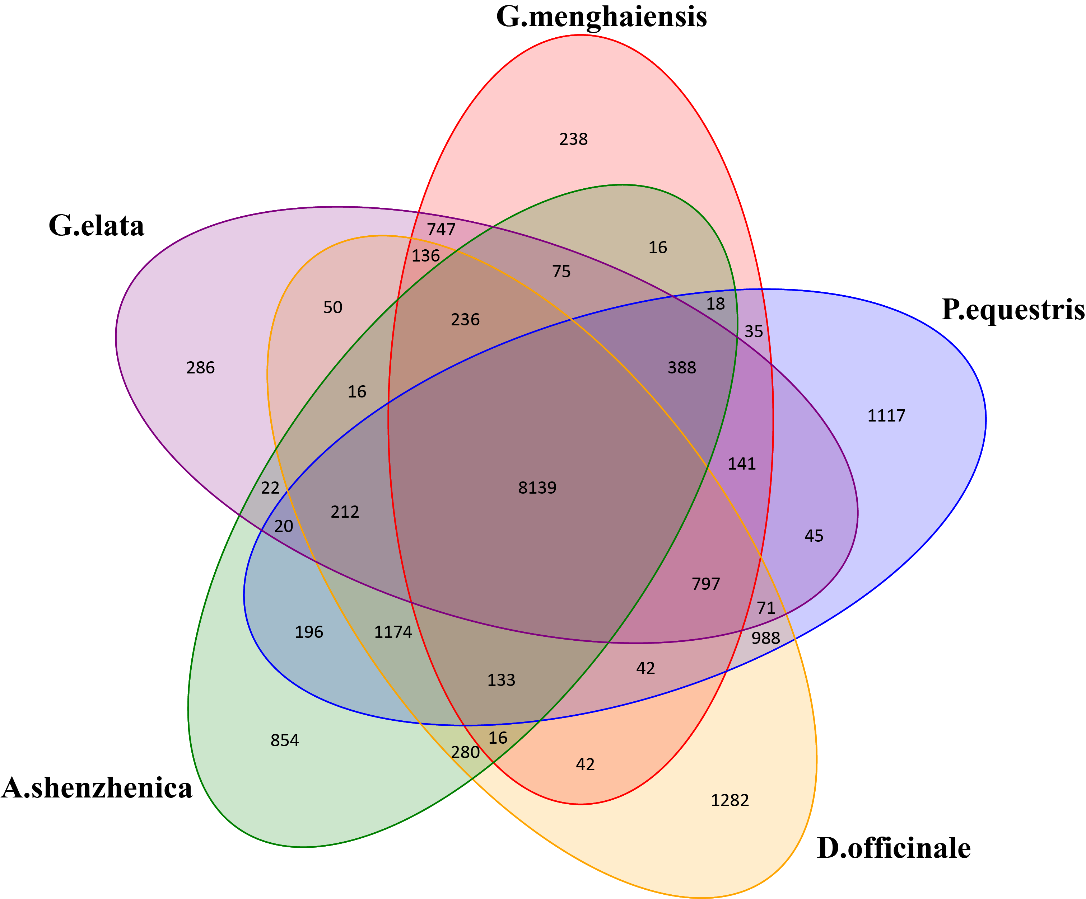


**Supplementary Figure S6. Common and unique gene families in five species from five orchid species.** Five orchid species: *P. equstris*, *Phalaenopsis equestris*; *A. shenzhenica*, *Apostasia shenzhenica*; *D. officinale*, *Dendrobium officinale*; *G. elata*, *Gastrodia elata*; *G. menghaiensis*, *Gastrodia menghaiensis*.
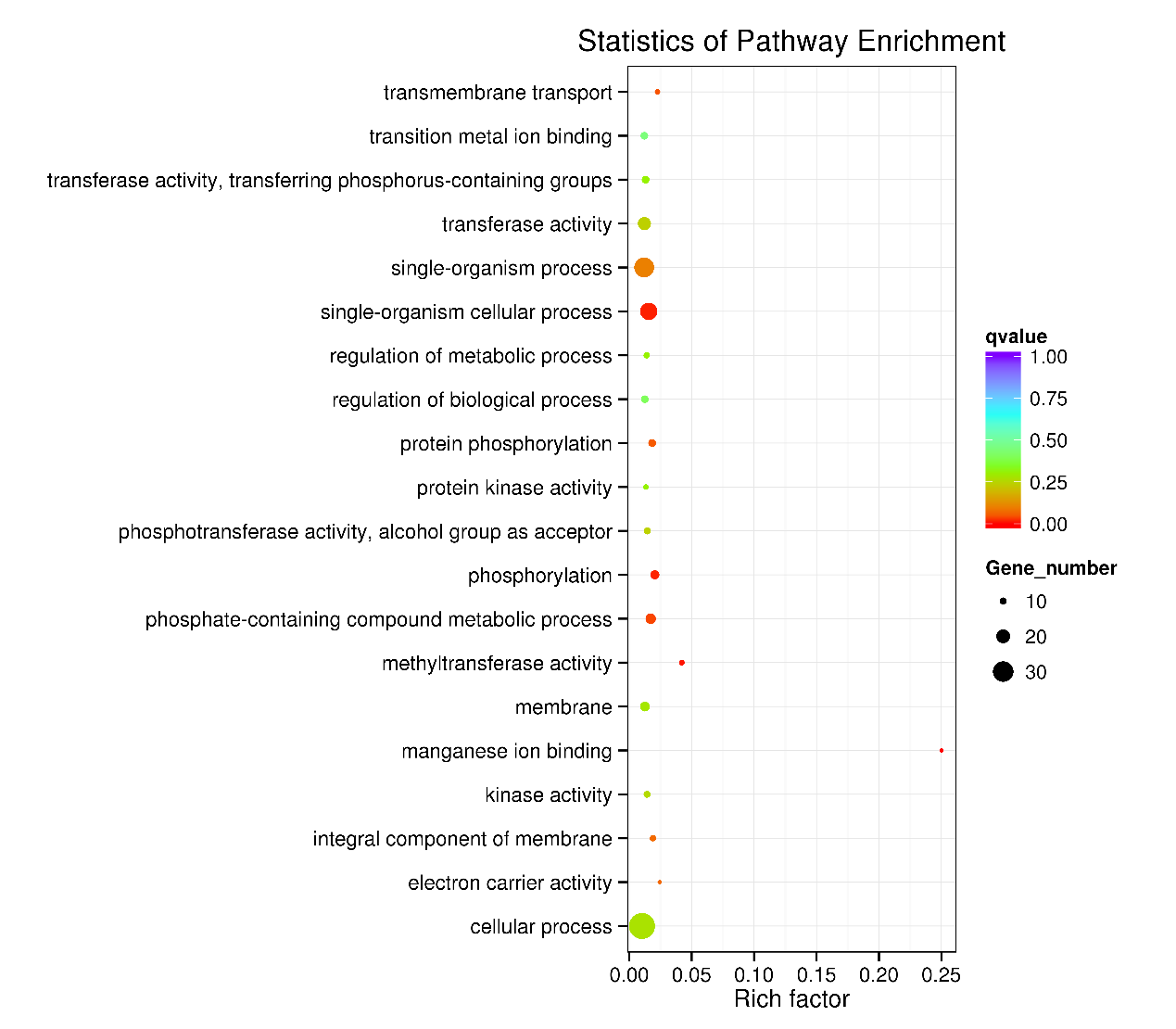


**Supplementary Figure S7. GO enrichment of unique gene families of *Gastrodia menghaiensis.*** Including regulation of cyclin-dependent protein serine/threonine kinase activity, potassium channel activity, potassium ion transmembrane transport, nutrient reservoir activity.


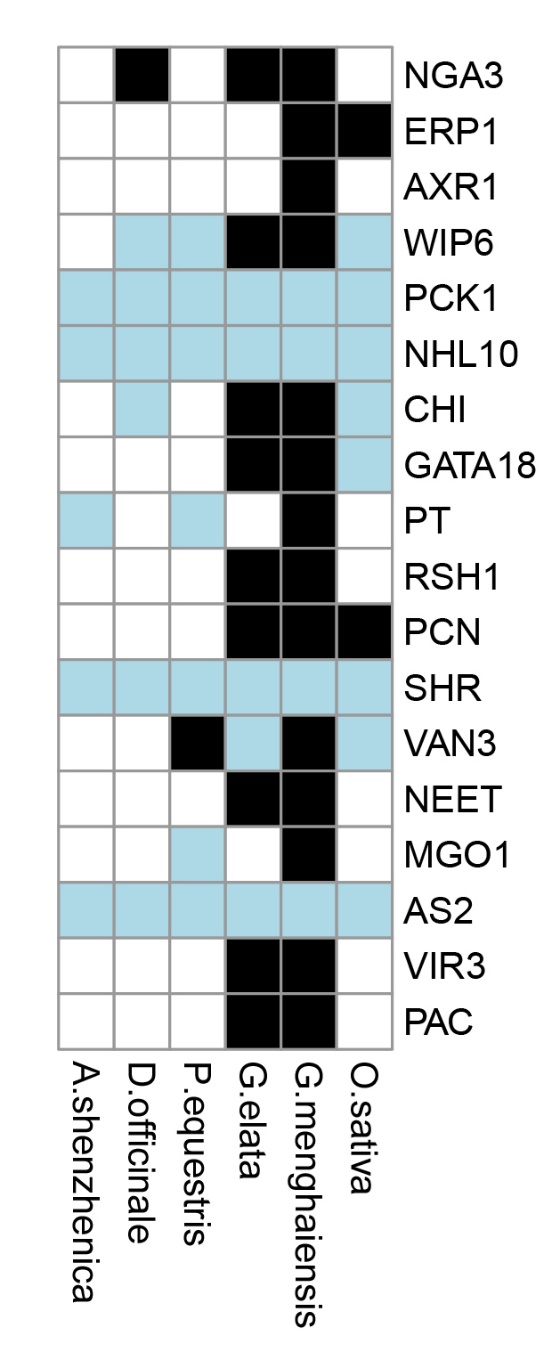


**Supplementary Figure S8. Genes involved in leaf development in six species.** Intact genes are indicated by white boxes, pale blue boxes indicating functional losses, black boxes indicating the physical losses. Six species: *O. sativa*, *Oryza sativa*; *P. equstris*, *Phalaenopsis equestris*; *A. shenzhenica*, *Apostasia shenzhenica*; *D. officinale*, *Dendrobium officinale*; *G. elata*, *Gastrodia elata*; *G. menghaiensis*, *Gastrodia menghaiensis*.


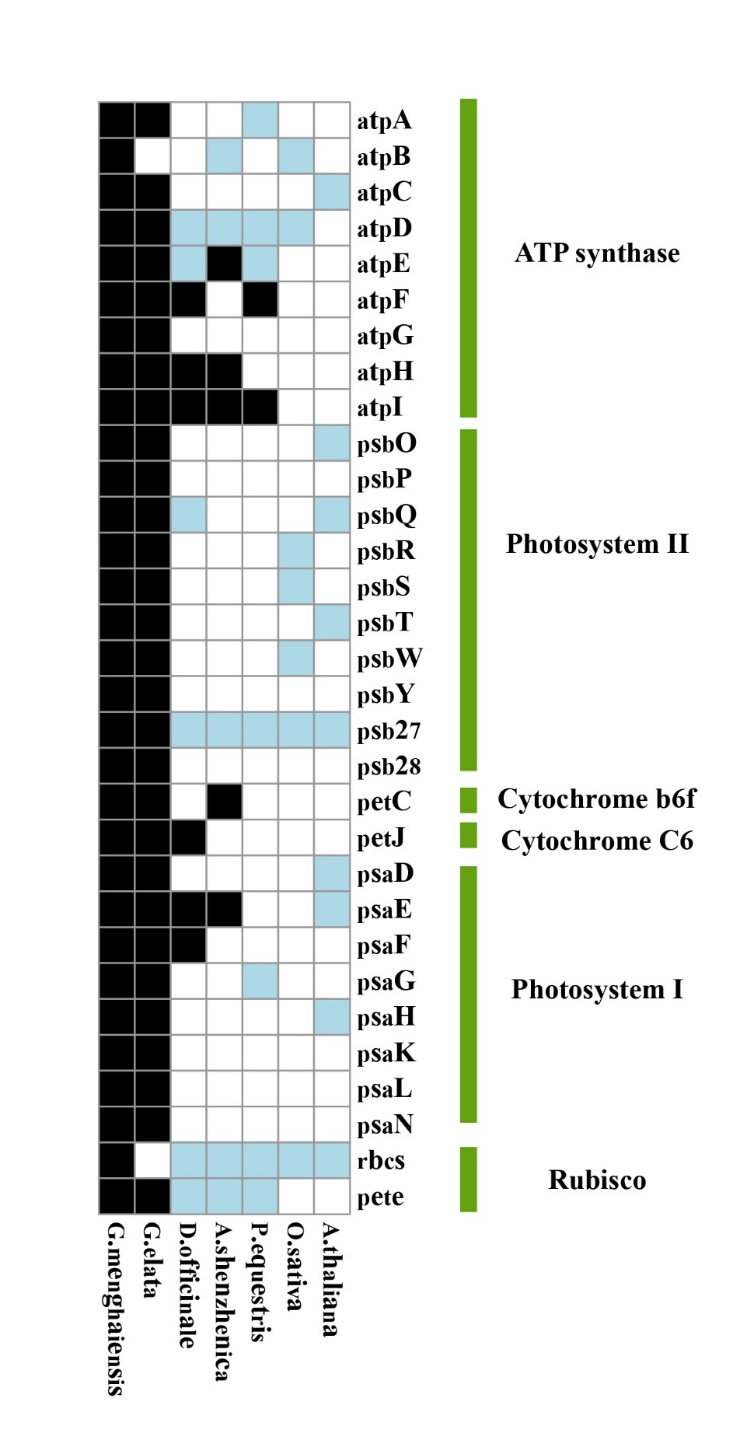


**Supplementary Figure S9. The presence of plastid encoded photosynthesis-related genes in seven species .** *A. thaliana*, *Arabidopsis thaliana*; *O. sativa*, *Oryza sativa*; *P. equstris*, *Phalaenopsis equestris*; *A. shenzhenica*, *Apostasia shenzhenica*; *D. officinale*, *Dendrobium officinale*; *G. elata*, *Gastrodia elata*; *G. menghaiensis*, *Gastrodia menghaiensis*). Intact genes are indicated by white boxes, whereas pale blue boxes indicating functional losses, black boxes indicating the physical losses.


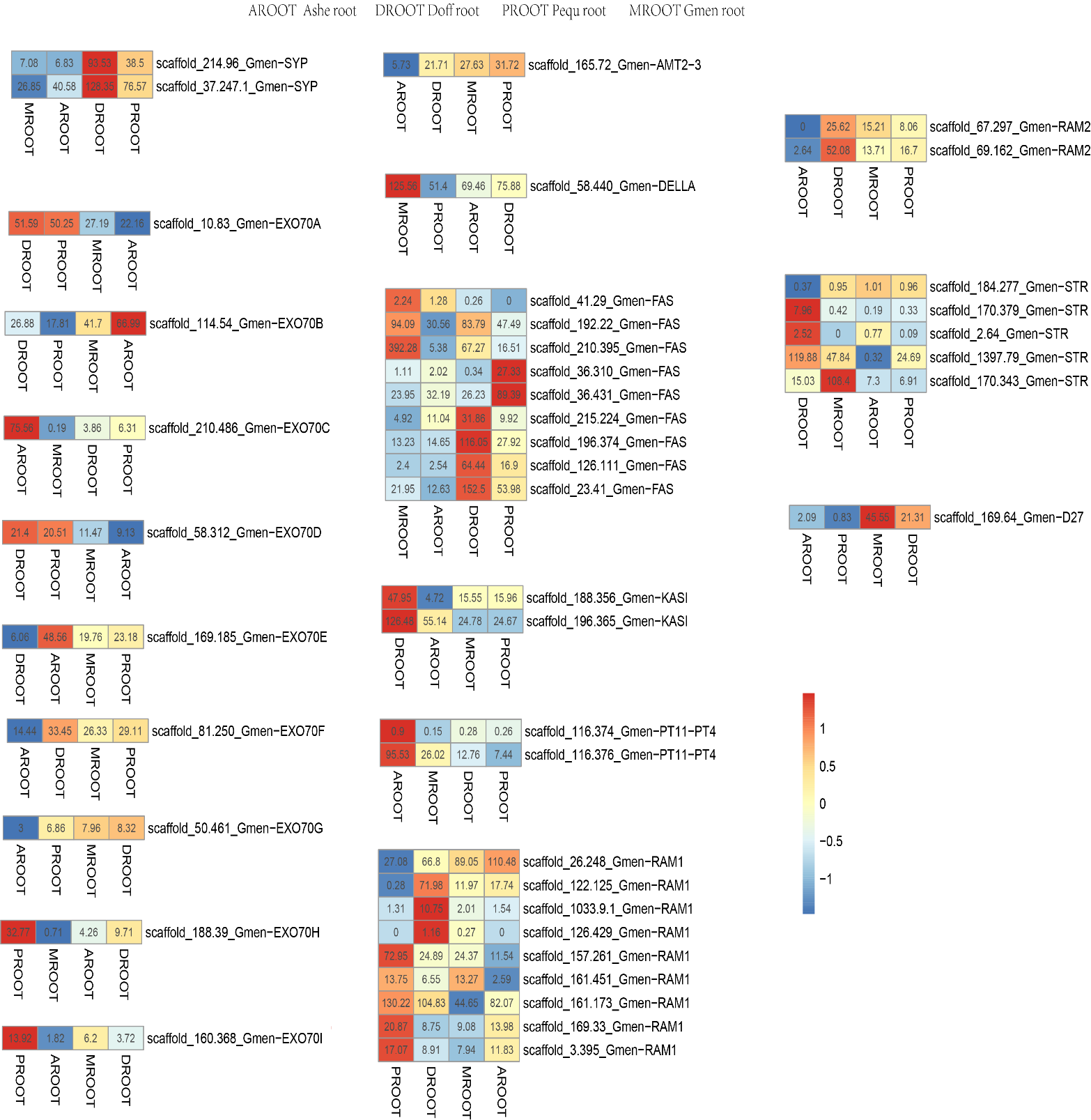


**Supplementary Figure S10. Transcriptome data of genes involved in the biointeraction between mycorrhizal fungi and orchids.**

*FAS*, fatty acid synthase; *KASII*, ketoacyl-ACP synthase II; *RAM1*, encoding a GRAS transcription factor; *RAM2*, encoding a Glycerol-3-Phosphate Acyl Transferase (GPAT); *PT11-PT4*, Phosphorus transporter; *AMT1, AMT2*, ammonium transporters 1 and 2. Genes related to colonization of mycorrhizal fungi (*SYP, Exo70A, Exo70B, Exo70C, Exo70D, Exo70E, Exo70F, Exo70E, Exo70H, Exo70I*); *D27*, related to strigolactone synthesis of *G. menghaiensis*. AROOT, roots of *Apostasia shenzhenica*; MROOT, roots of *Gastrodia menghaiensis*; DROOT, roots of *Dendrobium officinale*; PROOT, roots of *Phalaenopsis equestris*.
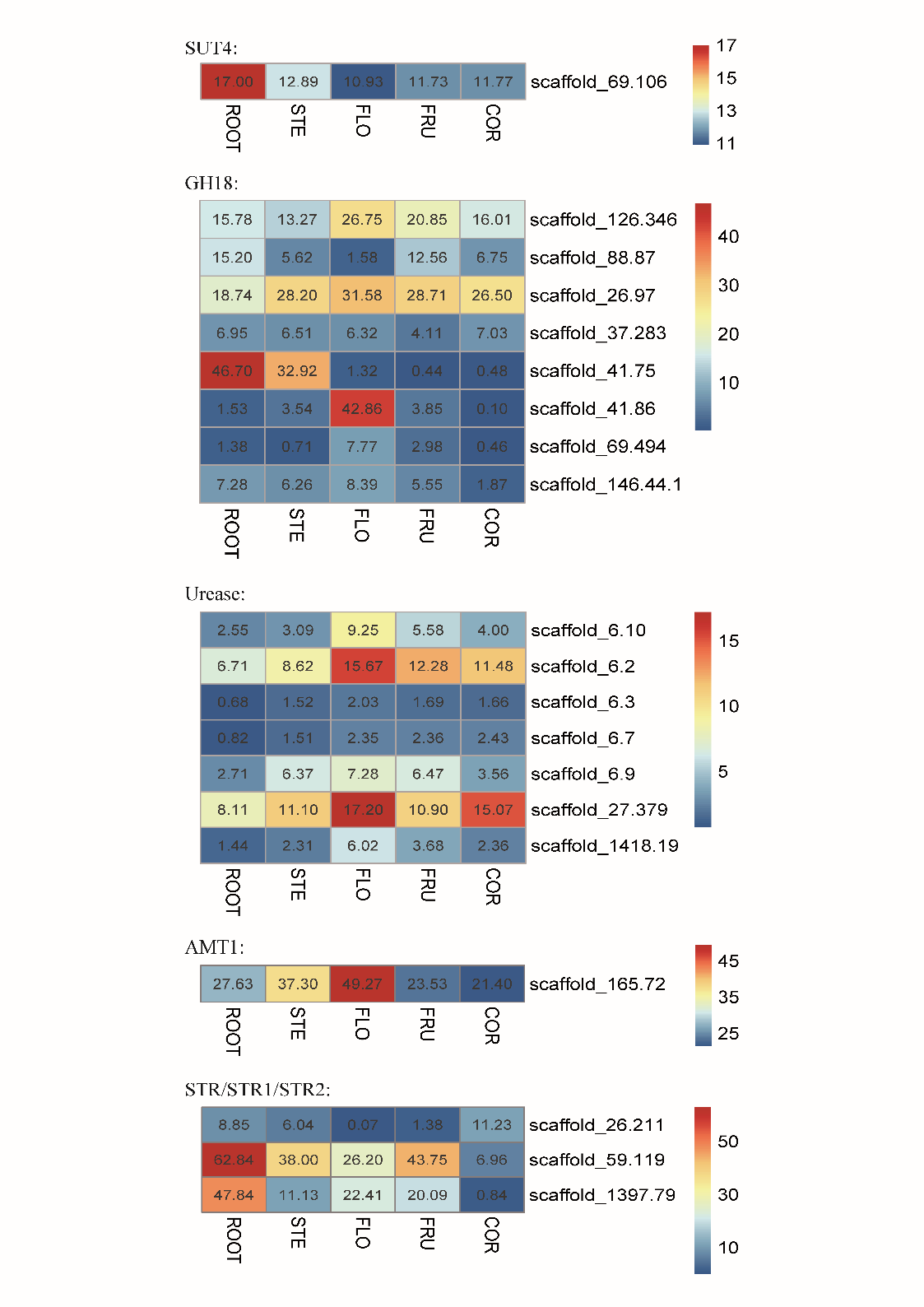


**Supplementary Figure S11. Transcriptome data of SUT4, GH18, Urease, AMT1, STR/STR1/STR2**

SUT4, sugar transporter 4; GH18, chitinase; AMT1, ammonium transmembrane transporter; STR/STR1/STR2, ABC transporter G family member 11; COR, corms; FRU, fruits; FLO, flowers; STE, stems; ROOT, roots.


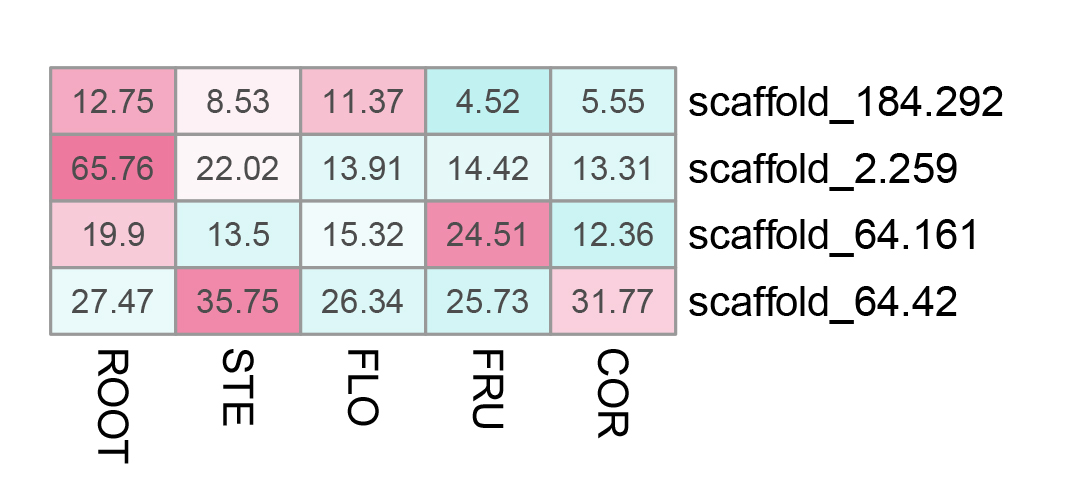


**Supplementary Figure S12.** Transcriptome data of four LysM-RLK in the *G. menghaiensis* genome. COR, corms; FRU, fruits; FLO, flowers; STE, stems; ROOT, roots.


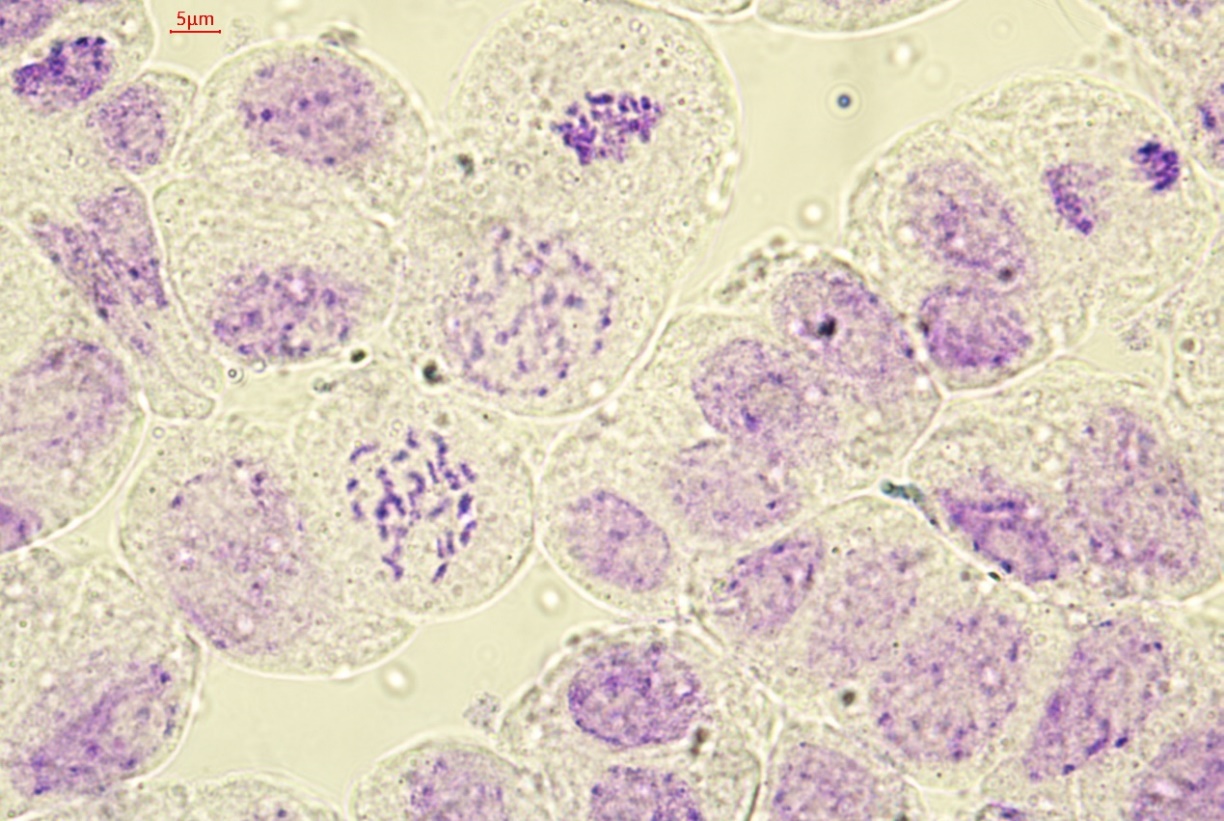
**Supplementary Figure S13. The number of chromosomes of *G. menghaiensis*.**

**REFERENCES**

1. Kanehisa M, Goto S, Kawashima S, Okuno Y, Hattori M: **The KEGG resource for deciphering the genome**. *Nucleic Acids Research* 2004, **32**:D277-D280.

2. Mao XZ, Cai T, Olyarchuk JG, Wei LP: **Automated genome annotation and pathway identification using the KEGG Orthology (KO) as a controlled vocabulary**. *Bioinformatics* 2005, **21**(19):3787-3793.
